# Carbon Gels–Green Graphene Composites as Metal-Free Bifunctional Electro-Fenton Catalysts

**DOI:** 10.3390/gels9080665

**Published:** 2023-08-17

**Authors:** Lilian D. Ramírez-Valencia, Esther Bailón-García, Adriana I. Moral-Rodríguez, Francisco Carrasco-Marín, Agustín F. Pérez-Cadenas

**Affiliations:** Materiales Polifuncionales Basados en Carbono (UGR-Carbon), Dpto. Química Inorgánica-Unidad de Excelencia Química Aplicada a Biomedicina y Medioambiente-Universidad de Granada (UEQ-UGR), ES18071 Granada, Spain; estherbg@ugr.es (E.B.-G.); aimr17@correo.ugr.es (A.I.M.-R.); fmarin@ugr.es (F.C.-M.)

**Keywords:** Electro-Fenton, xerogels, graphene, heteroatoms, metal-free, carbon-based materials

## Abstract

The Electro-Fenton (EF) process has emerged as a promising technology for pollutant removal. However, the EF process requires the use of two catalysts: one acting as an electrocatalyst for the reduction of oxygen to H_2_O_2_ and another Fenton-type catalyst for the generation of ·OH radicals from H_2_O_2_. Thus, the search for materials with bifunctionality for both processes is required for a practical and real application of the EF process. Thus, in this work, bifunctional electrocatalysts were obtained via doping carbon microspheres with Eco-graphene, a form of graphene produced using eco-friendly methods. The incorporation of Eco-graphene offers numerous advantages to the catalysts, including enhanced conductivity, leading to more efficient electron transfer during the Electro-Fenton process. Additionally, the synthesis induced structural defects that serve as active sites, promoting the direct production of hydroxyl radicals via a 3-electron pathway. Furthermore, the spherical morphology of carbon xerogels enhances the accessibility of the reagents to the active sites. This combination of factors results in the effective degradation of Tetracycline (TTC) using metal-free catalysts in the Electro-Fenton process, achieving up to an impressive 83% degradation without requiring any other external or additional catalyst.

## 1. Introduction

The contamination of water sources is a latent environmental problem, especially when polluting substances are not controlled and are detrimental to the health of an entire ecosystem. One of the most concerning groups of emerging pollutants is active pharmaceuticals due to their highly harmful nature. These substances are continuously being released into various aquatic environments, primarily because of the rapid expansion of the pharmaceutical industry. Unfortunately, traditional wastewater treatment methods have been ineffective in eliminating these pollutants, further exacerbating the issue. The persistence of active pharmaceuticals in the environment poses significant challenges to ensuring water quality and environmental health [1,2]. Therefore, it becomes a priority to focus on finding the most efficient form of treatment to remove these persistent substances from the water.

Advanced oxidation processes (AOPs) are so far from the most promising treatment for the near-total degradation of pharmaceutical contaminants [3] since they are based on the generation of strongly oxidizing and reactive species such as the hydroxyl radical (*OH) that are capable of degrading organic contaminants to simple compounds that are, ideally less toxic, being able, in some cases, to reach complete mineralization, i.e., obtaining water, CO_2_ and inorganic salts [4]. In addition, these hydroxyl radicals can be obtained in situ using different AOPs processes such as Fenton, Electro-Fenton and photo-Fenton using H_2_O_2_, ozonolysis using O_3_, and UV (ultraviolet light), among others. In comparison to reagents like O_3_ and the risks of UV utilization, Fenton-based processes offer a more cost-effective solution. These processes present various strategies to reduce expenses while maintaining simplicity, low toxicity, and excellent performance [5].

In principle, the Fenton process can be carried out at room temperature and under atmospheric pressure and consists of reacting a mixture of H_2_O_2_ and Fe^2+^, as shown in (Equation (1)), all in an acid medium (pH 3), generating hydroxyl radicals capable of degrading a wide range of water contaminants [6,7,8]. At the same time, Fe^2+^ ions can be regenerated (Equation (2)) by reducing Fe^3+^ species with excess H_2_O_2_ and producing perhydroxyl radicals (HO_2_*), which are also oxidizing but are weaker than hydroxyl radicals [9]. Therefore, it may be a cyclic mechanism, but not entirely efficient for three main reasons. First, the reaction rate of H_2_O_2_ and Fe^2+^ is much faster than the reduction of Fe^3+^ to Fe^2+^, which limits the generation of hydroxyl radicals since iron sludge is formed due to the accumulation of the Fe^3+^ species that precipitates in the form of iron oxides, making treatment difficult with additional separation processes [10,11]. Second, the use of H_2_O_2_ may represent another barrier because although it is a readily available reagent, its handling and storage are hazardous. Thirdly, the efficacy of Fenton reagents (comprising iron and H_2_O_2_) hinges on operating within a specific pH range of 3 to 4 to achieve optimal catalytic activity. When used outside this optimal range, their efficiency diminishes due to the occurrence of iron precipitation and the degradation of H_2_O_2_ into O_2_ and H_2_O [12]. This represents an impractical situation since not all wastewater to be treated has this pH range which would imply its adjustment, representing an increase in process costs [10].

(1) $${\mathrm{Fe}}^{2+}+H_{2}O_{2}\to {\mathrm{Fe}}^{3+}+{\mathrm{OH}}^{-}+*\mathrm{OH}$$

(2) $${\mathrm{Fe}}^{3+}+H_{2}O_{2}\to {\mathrm{Fe}}^{2+}+{\mathrm{HO}}_{2}^{*}+H^{+}$$

Given the information provided, Electro-Fenton emerges as a promising technology that combines various strategies to overcome the obstacles encountered when using traditional Fenton. One of the primary issues addressed is the leaching of Fe^3+^ species, which can be minimized by incorporating a robust Fenton phase primarily supported on carbonaceous materials [13,14], which allows reactive species to mainly form on the surface of the catalyst, facilitating their recovery, reuse and control of the kinetics of oxidative degradation [15]. In addition, they enable increasing the pH operating range, working at a pH close to neutral [16,17,18]. It is also a process that gives way to the in situ electro-generation of H_2_O_2_, which in turn allows the formation of hydroxyl radicals and also favors the continuous and more efficient regeneration of Fe^2+^, reducing the costs and problems involved in the direct use of H_2_O_2_, as well as improving catalytic activity by avoiding the accumulation of Fe^3+^ and thus the formation of sludge [19,20].

Thus, the reaction mechanism of the Electro-Fenton process involves two steps that are determinant in the efficiency of the process and for which the use of two types of catalysts is required. The first step is the in situ generation of H_2_O_2_, which is carried out by means of O_2_ electro-reduction (ORR); here, it is necessary to develop catalysts that allow a high selectivity toward the transfer of two electrons and not four electrons since, in the latter case, H_2_O and not H_2_O_2_ would be obtained. The other decisive step is the transformation of H_2_O_2_ to the hydroxyl radical, for which another type of catalyst with a Fenton-type active phase is also required to carry out this reaction. However, it is much more interesting to focus on the development of catalysts that allow this dual functionality to be carried out.

The use of carbon-based materials would become an excellent option due to their low cost, high electronic conductivity, abundance, good chemical stability, and tunable surface, textural, and chemical properties [21], in which different types of active phases can be introduced; they have even been found to be efficient catalysts for ORR via two electrons [22,23,24]. Within carbon-based materials, carbon gels are good candidates for catalytic applications, including Fenton-type processes [25], oxygen reduction [26] and CO_2_ reduction electrocatalysts [27] due to their tunable textural and chemical characteristics, which can be practically tailored from the synthesis conditions [28], doped with nitrogen [29], even provoking the formation of amazing metal-based nanostructures [30].

Therefore, carbonaceous materials can catalyze the electro-reduction of O_2_ to H_2_O_2_ and parallel the supported Fenton-type active phases on carbon, increase the production of *OH radicals and, consecutively, enhance the efficiency of degradation via Electro-Fenton. Different metallic phases such as Fe, Cu, Co, Ce, Mn, Mo and Ni have been used, forming a carbon composite, either monometallic, bimetallic or trimetallic, in all cases presenting a high Fenton activity [31,32,33,34]. Most of the synthesis methodologies for this type of material consist of encapsulating these metallic particles in the carbon core/shell structures so that, in this way, the activity can be modulated. Luo and co-authors [35] synthesized core/shell nanoparticles of Cu-doped Fe@Fe_2_O_3_. Their findings indicated that Cu^0^ and Fe^0^ underwent reactions with O_2_, resulting in the production of H_2_O_2_. Furthermore, Fe^2+^ and Cu^2+^ were found to be responsible for generating *OH radicals through the utilization of in situ generated H_2_O_2_. On the other side, Ghasemi and co-authors [36] took advantage of the high conductivity of carbon nanotubes to develop a cathode consisting of carbon nanotubes and CuFe nano-layered double hydroxide. The investigation demonstrated that the inclusion of CNTs led to an enhancement in H_2_O_2_ generation, while the atoms of both Cu and Fe played a pivotal role in facilitating the generation of *OH radicals. Sun and co-authors [37] also used CNTs in conjunction with a compound of CoFe alloy/N-doped carbon (CoFe@NC), which were used to modify a nickel foam and then used as a cathode. This research also found that CNTs produce in situ H_2_O_2_, while activation to *OH radicals was facilitated by (CoFe@NC). In addition to being able to modulate the activity in this type of bifunctional catalyst, it is necessary to take certain factors into account. The carbonaceous materials used must have an adequate porosity, which facilitates the effective diffusion of O_2_ molecules. Additionally, having a high surface area is crucial to provide an ample number of active sites. Moreover, ensuring high conductivity is essential for the optimal performance of the electrode. By meeting these criteria, the electrode can maximize its efficiency and effectiveness. For example, recently Qi and co-authors [38] took advantage of the large specific surface area and large pore volume of etched graphite felt (EGF) to obtain enough active sites to improve the performance of the Cu-doped Fe_2_O_3_ nanoparticles/EGF electrode. On the other hand, Cao and co-authors [39] synthesized nitrogen-doped hierarchically porous carbon with embedded iron oxide particles with pores, including abundant micropores with a narrow diameter of around 0.7 nm and mesopores with a diameter of 3.5 nm, the strong interaction between these pores and doped nitrogen could improve the selectivity for H_2_O_2_ generation, exhibiting excellent performance for phenol degradation. In addition to these parameters, the materials are also required to be highly conductive to facilitate electron transfer. Xie and co-authors [40] employed reduced graphene oxide synthesized via the Hummer method to improve the conductivity of metal–organic frameworks (MOFa), obtaining a bifunctional catalyst with improved charge transfer.

Nonetheless, a persisting challenge remains in the form of catalysts detachment and metal leaching. This issue leads to the deactivation of the cathode and secondary pollution due to the release of leaching ions. In view of this, recently, in a few works, different carbon materials have been doped with heteroatoms such as O and N, which can serve the function of a Fenton-type catalyst and catalyze the reaction of H_2_O_2_ to hydroxyl radicals [41,42,43,44]. This approach emerges as a viable alternative that circumvents the need for using metals, thereby potentially overcoming the drawbacks associated with metal-based systems. Yang and co-authors [43] synthesized electrochemically exfoliated graphene (EEGr), doping it with nitrogen using ammonium nitrate, found that at a 1:1 ratio of EEGr/ammonium nitrate, it has a significant electrocatalytic performance, converting the generated H_2_O_2_ into hydroxyl radicals. This same author [45] then proposed co-doping of EEGr with N and S, which significantly improved the percentage of degradation of the contaminant under study (phenol). Qin and co-authors [42] doped commercial carbon nanotubes CNTs with oxygen by means of acidizing treatment with concentrated HNO_3_ and H_2_SO_4_ solution, which served as a bifunctional catalyst to significantly promote the ability of H_2_O_2_ production and activation to *OH for degrading phenol. Haider y co-authors [46] fabricated N-doped carbon nanofibers derived from the carbonization of polyaniline (PANI), the bifunctional activity of this type of catalyst is attributed to the N-graphitic content, which enhances the generation of H_2_O_2_, while the N-pyridinic content generates the *OH radicals.

However, tedious, expensive and toxic synthesis processes are necessary to obtain many of these materials, i.e., nanotubes, graphene or nanofibers, also metal-based materials or two catalysts for ORR and Fenton processes are still required. In this work, carbon xerogel microspheres doped with green graphene (eco-graphene) and nitrogen have been proposed as metal-free bifunctional Electro-Fenton catalysts. These new materials have the novelty that is completely free of metal and present high activity for the direct production of OH* radicals, avoiding the use of two catalysts in the EF process. The micro and mesoporous structure of these carbon xerogels will facilitate the mass transfer in the electro-reaction. Furthermore, the spherical morphology of the gels will allow the surface to be fully exposed to the electrolyte and consequently can be more accessible to the reagents. Eco-graphene doping will enhance the electrical conductivity of these materials. One of the samples has also been doped additionally with nitrogen in order to see the role of this element in this type of material. It should be noted that the synthesis of green graphene consists of a clean process in which no toxic residues are used or generated, as is normally observed when synthesizing graphene using the Hummer method since the synthesis is carried out via a hydrothermal method, and glucose is used as starting material [47]. The influence of all these characteristics of the developed carbon gels has been evaluated in oxygen electro reduction (ORR). Their performance as bifunctional metal-free Electro-Fenton catalysts was evaluated in the degradation of tetracycline, an antibiotic in common use and detected frequently in water, all this within a one-pot process.

## 2. Results and Discussion

### 2.1. Morphological and Textual Characterization

**Figure 1:**
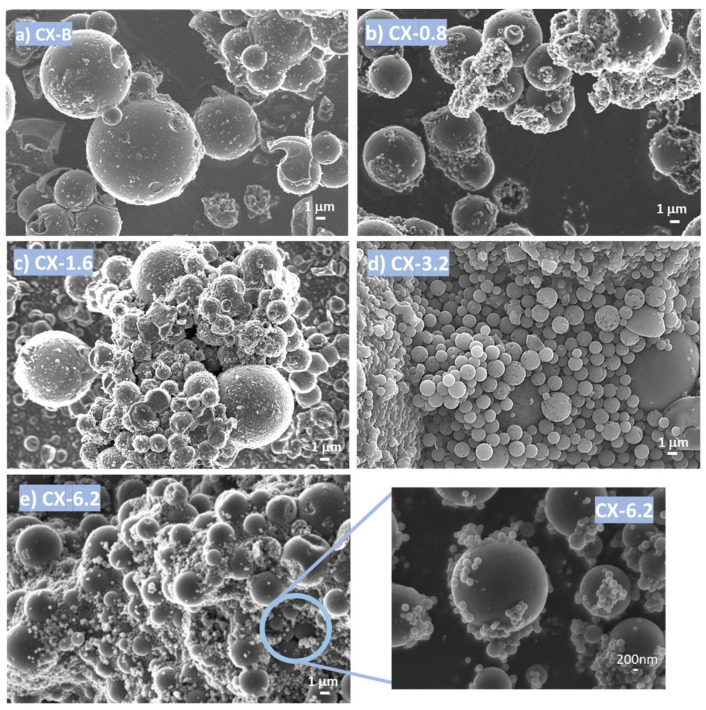
SEM images of pure xerogel (CX-B) and eco-graphene doped (CX-Y) carbon samples at a magnification of 2.8 Kx. (7.0 Kx for the CX-6.2 magnified image).

SEM (Figure 1) reveals that the samples are composed of agglomerates of adjacent microspheres that form voids between them; therefore, the materials are expected to be meso-macroporous in addition to having microporosity consisting mainly of pores within the spheres. It is interesting how the samples doped with eco-graphene are formed of spheres with different size ranges, as a mixture of small, medium, and larger spheres; this characteristic was more pronounced in the sample with a higher percentage of eco-graphene (Figure 1e). While the sample without eco-graphene is formed by bigger spheres with a more uniform size but more irregularly shaped, so it may be that the introduced eco-graphene influences the polymerization process and, therefore, the final texture of the doped materials even at low percentages.

**Figure 2:**
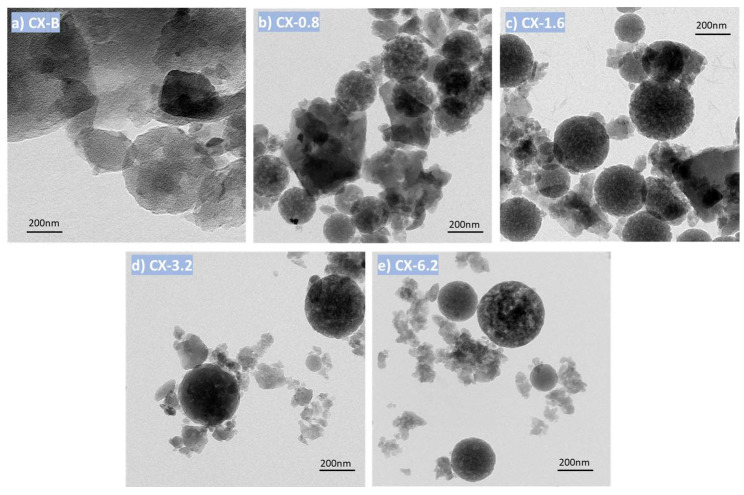
HRTEM images of pure xerogel (CX-B) and eco-graphene-doped (CX-Y) samples.

**Figure 3:**
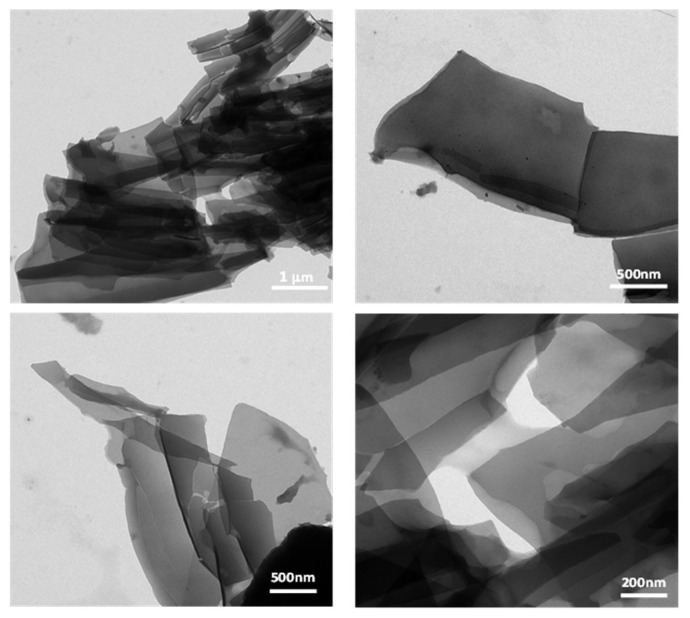
HRTEM images Eco-Graphene from glucose synthesized by hydrothermal synthesis.

The same conclusions can be obtained via transmission electron microscopy (TEM), Figure 2. Again, bigger particles are observed in non-doped samples (CX-B), whereas spheres around 285 nm are observed in samples doped with eco-graphene. While the carbon xerogel without eco-graphene shows no uniform spheres and parts of larger spheres are observed, more defined spheres are observed in eco-graphene-doped samples. It is important to highlight that some eco-graphene sheets are distributed in close contact around the spheres in all eco-graphene-doped samples. On the other hand, the synthesized eco-graphene (Figure 3) is composed of smooth and apparently defect-free nanosheets.

**Figure 4:**
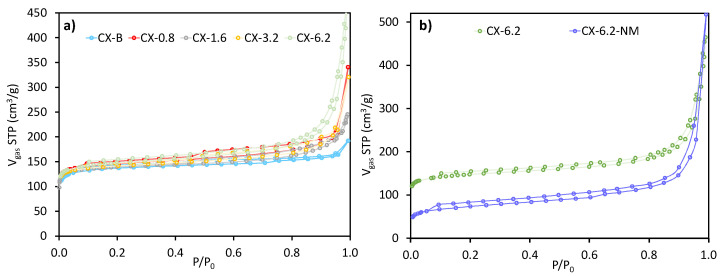
N_2_-adsorption/desorption isotherms of (**a**) doped and undoped eco-graphene samples, (**b**) doped and undoped nitrogen samples.

The textural properties of the xerogels have been analyzed by N_2_ and CO_2_ adsorption isotherms at −196 °C and 0 °C, respectively. N_2_ adsorption–desorption isotherms are collected in Figure 4, while Table 1 summarizes the results obtained from the analysis of both type of isotherms. 

In all samples, the presence of micropores is evident because at very low relative pressures, there is a high N_2_ adsorption. The isotherms have a similar shape, showing a type I isotherm according to the IUPAC classification. However, the samples doped with eco-graphene show a much more rapid increase in N_2_ adsorption at high relative pressures close to 1, which could be indicative of the presence of mesoporosity [48,49] due to the interparticle voids. The higher the eco-graphene content, the higher the N_2_ adsorption increase at P/P_0_, near 1. From Table 1, the presence of graphene clearly favours mesoporosity development (V_meso_), while the microporosity seems to be unaffected (W_0_ and L_0,_ both with CO_2_ and N_2_). In addition, the influence on the textural properties of nitrogen doping (Figure 4b) provokes a loss of microporosity, blocking the wider micropores.

### 2.2. Raman Spectroscopy

Raman spectroscopy was employed to determine the degree of graphitization of samples; for this purpose, the intensities of the D and G bands (I_D_ and I_G_) were identified, which represent the degree of defects or graphitization, respectively, in carbon structures. Therefore, the I_D_/I_G_ ratio is considered an important parameter to indicate the ordering of the materials; when there is a high degree of graphitization, this ratio will be much lower [50].

**Figure 5:**
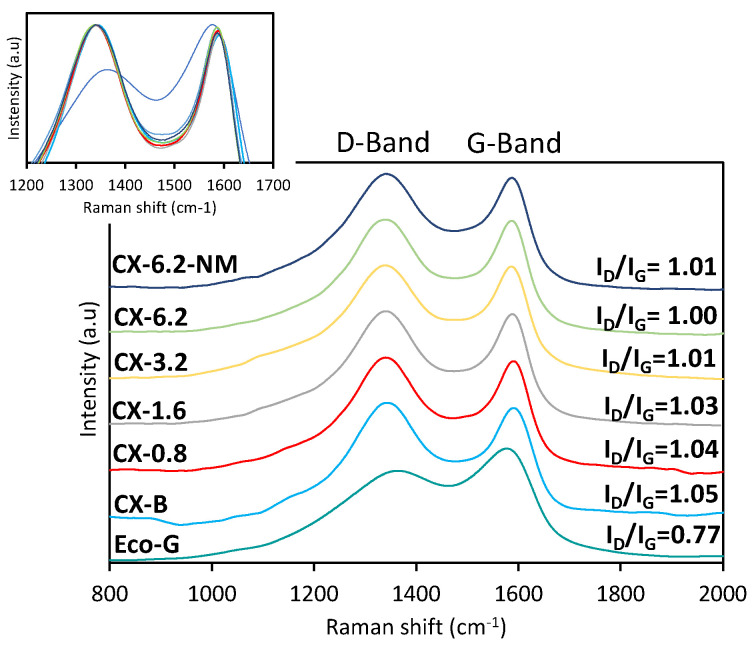
Raman spectra of pure CX-B, Eco-graphene-doped carbon samples, N-doped carbon samples and pure Eco-graphene.

The I_D_/I_G_ ratio of all samples is presented in Figure 5. It was observed that as the percentage of eco-graphene increases, the intensity of the G-band also increases with respect to the intensity of the D-band. On the other hand, the D-band of the starting eco-graphene presents a lower intensity, and the I_D_/I_G_ ratio is consequently the lowest, which would indicate a very low ratio of defects/high graphitization. This point represents a great advantage because by means of the hydrothermal synthesis and using glucose as raw material, a much more reduced and less defective graphene was obtained compared to the graphene obtained via the Hummer method. Due to its synthesis nature, strong oxidizing agents are employed to obtain graphene sheets with more defects [51].

A noticeable rightward shift in the G-band is observed in the eco-graphene doped samples with respect to the G-band of the original eco-graphene, possibly attributed to the nature of the new carbon–carbon composite formed between graphene and the xerogel [52]. The introduction of nitrogen species increases the defects in the material, so the I_D_/I_G_ ratio increases slightly.

### 2.3. Analysis Elemental and XPS

The total amount of nitrogen, carbon and hydrogen was obtained via elemental analysis (Table 2). The non-nitrogen functionalized materials doped with eco-graphene have a representative amount of nitrogen traces, especially those doped with a high percentage. The origin of this nitrogen can be explained via the synthesis method of the eco-graphene, since during the hydrothermal process at 270 °C, the CTAB molecules decompose, releasing nitrogen and hydrogen gases that interact with the graphitic structure causing the doping with nitrogen and the reduction of the oxidized structure, respectively [47,53]. On the other hand, the nitrogen content increases after melamine functionalization of the CX-6.2 sample (CX-6.2-NM), indicating that the incipient impregnation process was very effective.

**Figure 6:**
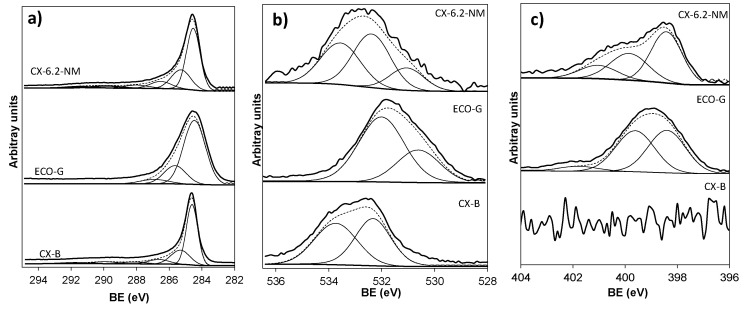
XPS patterns of the (**a**) C_1S_, (**b**) O_1S_ and (**c**) N_1S_ regions.

Regarding XPS, the C_1S_ spectra of the carbon microspheres (Figure 6a) were fitted to six peaks: C=C (284.6 eV), C-C (285.3 eV), C-O (286.6 eV), C=O (288.0 eV), O=C-OR (289.9 eV) and π-π* (291.7 eV) peaks. All fractions in this region are similar, indicating that undoped and both eco-graphene- and nitrogen-doped spheres have similar surface chemistry. In contrast, the C_1S_ spectra of eco-graphene only needed to be deconvoluted in three peaks: C=C (284.5 eV), C-C (285.7 eV), C-O (286.9eV). Pure Eco-graphene has a higher percentage of graphitic carbon C=C (Table 3) than carbon xerogel spheres. In the O_1S_ spectra, sample CX-6.2-NM had a significant change with respect to the pure carbon xerogel CX-B (Figure 6b); a new peak was identified that was absent in CX-B but present in pure eco-graphene. Therefore, this peak in the CX-6.2-NM sample was attributed to doping with eco-graphene and corresponded to quinones groups (530.7 eV) [54]. Quinone functional groups can serve as active sites for H_2_O_2_ production [55]. Note that in pure eco-graphene, the peak attributed to quinone groups represents 30.9% in comparison to CX-6.2-NM. The N_1S_ spectrum was also analyzed, finding that sample CX-B has no nitrogen functionalities, which agrees with what was seen through the use of elemental analysis. On the contrary, CX-6.2-NM and eco-graphene spectra were deconvoluted in three peaks: Pyridinic-N (398.4 eV), Pyrrolitic/Pyridonic (399.8 eV), graphitic-N (401.0 eV). In addition, sample CX-6.2-NM has a nitrogen content of 5%, a value very similar to that obtained in the elemental composition, indicating that doped nitrogen is homogeneously distributed along the surface. On the contrary, the eco-graphene presented a high surface amount of N of 11.3%.

### 2.4. Oxygen Reduction Reaction (ORR)

Oxygen electro-reduction was studied using a rotating disk electrode using 0.1 M KOH as the electrolyte. First, to analyze the effects of different chemical and textural properties on electrochemical behavior, cyclic voltammetry was performed.

**Figure 7:**
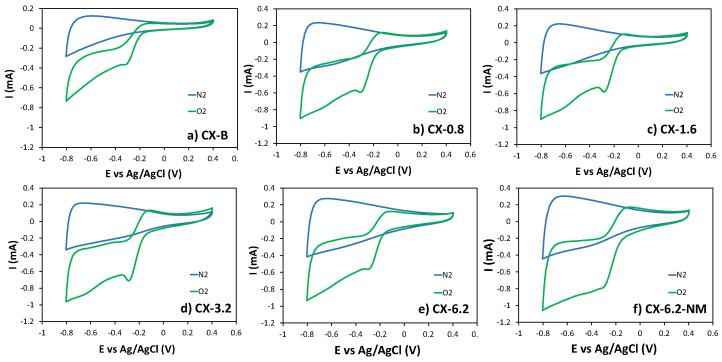
Cyclic voltammograms at 50 mV/s and 1000 rpm of (**a**) CX-B, (**b**) CX-0.8, (**c**) CX1.6, (**d**) CX-3.2, (**e**) CX-6.2, and (**f**) CX-6.2-NM in N_2_- and O_2_-saturated KOH solution.

The cyclic voltammograms in the presence of nitrogen and oxygen are plotted in Figure 7. It is observed that there is an increase in current intensity around −0.2 V vs. Ag/AgCl when the electrolyte is saturated with oxygen, indicating that all samples are active for ORR. However, some samples appear to be more active than others; therefore, it is necessary to determine to what extent the percentage of eco-graphene and doped nitrogen can influence the kinetic current density (Jk) in the ORR as well as the reaction mechanism by knowing the number of electrons transferred (n). 

**Figure 8:**
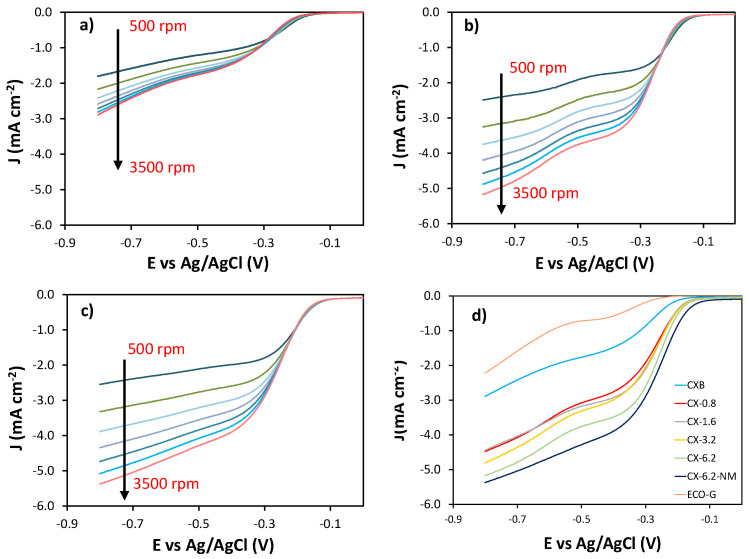
LSV at different rate speeds (**a**) CX-B, (**b**) CX-6.2, (**c**) CX-6.2-NM, and (**d**) LSV at 3500 rpm.

The above can be investigated in more detail using linear sweep voltammetry (LSV) at different rotation rates ranging from 500 rpm to 3500 rpm (Figure 8a–c). This speed sweep was performed in all the samples with the objective of decreasing the mass transfer limitations by increasing the electrode rotation speed so that as the revolutions per minute increase, and the current intensity increases as well. LSV comparison between samples was performed at the highest rotational speed (3500 rpm) (Figure 8d), finding that the presence of eco-graphene from the smallest percentage, 0.8%, already improves the catalytic activity. Increasing the percentage of eco-graphene, high activity is obtained; thus, the CX-6.2 sample showed the highest Jk (Table 4). This can be attributed to the enhancement of electron transfer provided by eco-graphene to carbon microspheres, allowing for improved ORR efficiency. The Jk increases even more after nitrogen doping. This high activity of the nitrogen-doped sample can be related to the majority content of N-pyridine species (Table 3), which serve as active sites, benefiting electron transfer and, thus, ORR [56]; this may also explain why the reaction onset potential (Table 4) in the nitrogen-doped sample decreases significantly with respect to the other eco-graphene doped and undoped samples.

**Figure 9:**
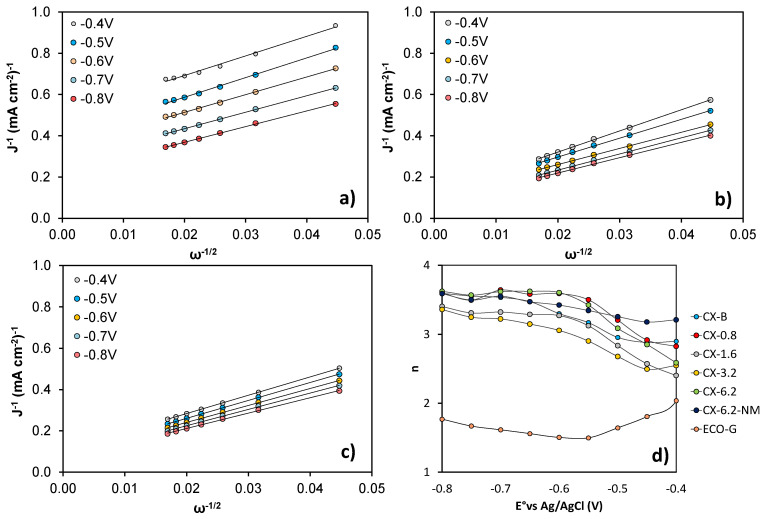
K-L plots of CX-B (**a**), CX-6.2 (**b**), and CX-6.2-NM (**c**) samples, and the number of electrons transferred at each potential for all the samples (**d**).

The LSV data obtained were subsequently fitted to the Koutecký–Levich equation to not only know the values of Jk but also to identify the reaction mechanism involved in the use of each of the synthesized materials. K-L plots at different potentials are represented in Figure 9a–c. All samples show first-order reaction kinetics, and the undoped sample (CX-B) has all the connecting lines completely parallel, indicating that there is no significant change in the electron transfer number of the ORR regardless of the potential. Conversely, a change in the slope from −0.4 V to −0.5 V is observed for the sample doped with 6.2% eco-graphene and nitrogen, indicating that the electron transfer increases as the potential increases.

This is confirmed by the analysis of the number of electrons transferred (Figure 9d). In general, all the samples have an electron transfer of around 3. It is known that a two-electron transfer is a typical mechanism for the in situ generation of hydrogen peroxide and its subsequent conversion to hydroxyl radicals in Electro-Fenton; however, it has been found earlier that a three-electron pathway is also possible [57]. Miao and co-authors [58] recently proposed a mechanism for this case, explaining that oxygen is reduced to H_2_O_2_ adsorbed, and it passes directly to form hydroxyl radicals without it having to desorb as normally occurs in a two-electron transfer (Equations (3) and (4)). In our case, the materials can have active sites able to carry out the reaction of Equation (3) but also have active sites capable of carrying out the reaction of Equation (4), all this when the potentials are lower than −0.6 V. When the applied potentials are greater than −0.6 V, the reaction in which O_2_ can be reduced to water via a four-electron pathway also comes into consideration.

(3) $$O_{2}\to O_{2\;ads}\to H_{2}O_{2\;ads}\overset{Catalysis\;1}{\to }\;H_{2}O_{2\;des}\;\}\;\;\;\;2e^{-}\;;\;H_{2}O_{2}{}_{des}\to H_{2}O_{2}{}_{ads}\overset{Catalysis\;2}{\to }*OH_{ads}\to *OH_{des}$$

(4) $$O_{2}\to O_{2\;ads}\overset{catalysis\;1}{\to }H_{2}O_{2\;ads}\overset{Catalysis\;1}{\to }*OH_{ads}\to *OH_{des}\;\;\;\;\}\;\;\;\;3e^{-}$$

**Figure 10:**
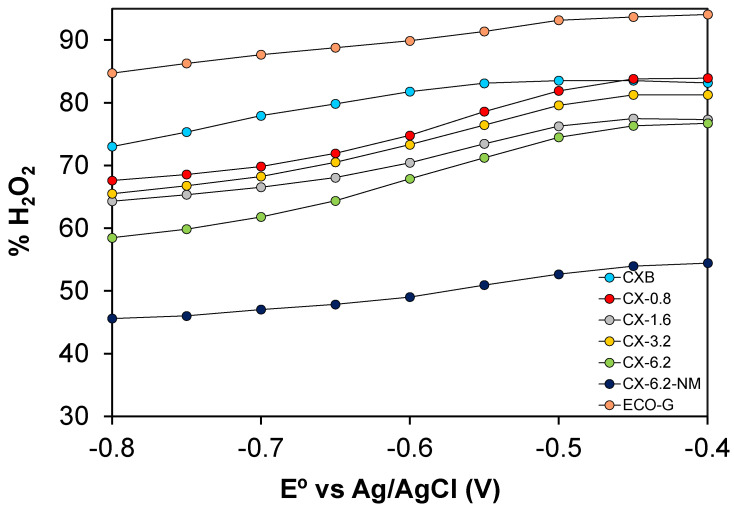
Production of H_2_O_2_ at each potential for all samples.

To corroborate the above, H_2_O_2_ generation was determined using a rotating disk electrode (Figure 10). For all samples, H_2_O_2_ production is observed to a greater or lesser extent depending on the potential. Confirming that at low potentials, the reaction via three-electron is not the only one to occur; if not, the production of desorbed H_2_O_2_ is also carried out, which will be used as an intermediate to obtain*OH radicals. On the other hand, at high potentials, it is observed that H_2_O_2_ production decreases, indicating that the selectivity changes toward water production; however, H_2_O_2_ is still being desorbed, indicating that the reaction of Equation (3) is also taking place.

The fact that the materials go through a three-electron pathway can be attributed to intrinsic carbon xerogel defects, which serve as active sites to rapidly adsorb O_2_ and accelerate the transformation of H_2_O_2_ to *OH radicals [42]. Moreover, their spherical morphology, as seen via SEM and TEM, allows these defects to be more exposed to the reagents. As such, the doping with eco-graphene does not influence the selectivity towards three electrons since, by itself, it goes toward a pathway of two (Figure 9d), while the undoped CX-B sample does go directly to three; so, in that sense, the eco-graphene only contributes in terms of improvement to electronic conductivity once doped in the materials since it has a lower catalytic activity by itself (Figure 9d).

Compiling the results, it is important to highlight that iron is a common Electro-Fenton catalyst par excellence that transforms the peroxide produced in situ to *OH radicals for the degradation of a pollutant. However, here we have synthesized metal-free carbon xerogel microspheres capable of not only producing H_2_O_2_ but also transforming it to *OH radicals (that is why a three-electron pathway is obtained). Moreover, as a further plus point, the presence of eco-graphene, especially when using 6.2% and nitrogen doping, improves the electronic conductivity of the samples. Therefore, the samples can be good candidates as bifunctional Electro-Fenton catalysts for the in situ generation of H_2_O_2_ and its transformation to *OH radicals, even directly to some extent.

### 2.5. Evaluation of Electrocatalysts for Electro-Fenton

**Figure 11:**
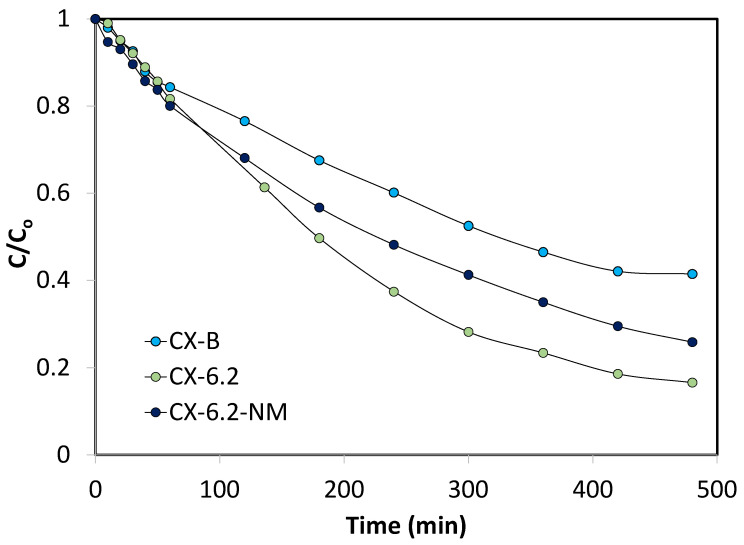
Normalized TTC concentration vs. time at potential of −0.6 V for undoped. microspheres, eco-graphene-doped microspheres, and N-eco-graphene-doped microspheres.

For the Electro-Fenton test samples, CX-6.2 and CX-6.2-NM were selected mainly for their high catalytic activity, as seen in ORR (Jk values Table 4); in addition, sample CX-B was also evaluated for comparison purposes. Tetracycline (TTC) was selected as a degradation target drug, and an average potential of −0.6V was used so that the selectivity was around three-electron combined with high activity. To attribute the degradation results clearly to the Electro-Fenton process, it was first necessary to eliminate the adsorption process by previously saturating the working electrode with the TTC at room temperature, in the dark, and finally setting the initial concentration at 15 ppm. From this point, the reaction was started, applying the selected potential with a constant O_2_ bubbling, observing the TTC degradation during the reaction time. These data are shown in Figure 11. All three samples were found to be able to degrade TTC to a greater or lesser extent; at this point, it is important to note that the degradation of tetracycline is entirely due to the fact that carbon xerogels can generate *OH radicals from the electro-reduction of O_2_ via three-electrons, since in one of our previous studies it was ruled out that TTC could be degraded by oxidation only with H_2_O_2_ or by a simple oxidation–reduction process upon the passage of the applied current [59].

Sample CX-B shows a degradation equal to 58%, which could be attributed to the electro-generation of *OH radicals using a three-electron pathway via the intrinsic defects of the carbon xerogel microspheres. The doping with eco-graphene increases the TTC degradation above 70%, both in N-doped and undoped. However, although sample CX-6.2-NM achieved high catalytic activity in ORR compared to the other samples (higher Jk) and the electron transfer is quite similar (around 3.4), and the percentage of TTC degradation (73%) was not higher than that obtained with sample CX-6.2 (83%), which would be ascribed to a higher participation of the four electron pathway and, thus, less hydroxyl radical production (competence between two and four e- pathway) as observed through the decreased amount of H_2_O_2_ produced and to the decreased active surface, which also plays an important role. Thus, the new N active site could act as an active site for ORR via the 4e route, thus increasing the current density but decreasing H_2_O_2_ and *OH hydroxyl radicals, and thus, TTC degradation. The introduction of new nitrogen active sites also decreases the surface area, decreasing the exposed surface for EF reaction. On the other hand, the high percentage of the degradation of sample CX-6.2 is due to its high selectivity toward the direct production of hydroxyl radicals together with the enhanced catalytic activity by incorporating eco-graphene into xerogel microspheres. 

Considering the obtained results, in futures works, the miniaturization of carbon-sphere size from micro- to nanospheres will be considered in order to favour the diffusion of reactant to the catalyst surface (O_2_ and pollutants) and more eco-friendly synthesis routes will be established (hydrothermal methods). Moreover, the co-doping of the sphere surface with different heteroatoms will also be performed in order to increase the catalytic activity in the EF process.

## *3.* Conclusions

Novel bifunctional metal-free catalysts for water decontamination using a one-pot Electro-Fenton process have been obtained. These catalysts based on carbon xerogel microspheres were synthesized via inverse emulsion, and the amount of eco-graphene introduced has been optimized to maximize the catalytic activity of O_2_ electro-reduction. These new materials are free of metal and present dual functionality for the direct production of OH* radicals, avoiding the use of two catalysts in the Electro-Fenton process.

The dual catalytic behavior seems to be related to the morphology and the structure of the carbon microspheres where would be sited the active sites. Very well-formed eco-graphene improves electrical conductivity, and nitrogen doping is not crucial in this process. These materials catalyze the O_2_ electro-reduction through a three-electron pathway in more or less extension depending on the applied potential. At lower potentials, both three- and two-electron pathways take place, both being catalytic pathways of hydroxyl radical generation responsible for the mentioned bifunctional behaviour. 

Tetracycline degradation was achieved in 83% using CX-6.2 material; this opens a very promising avenue for the development of carbon-based metal-free bifunctional materials; however, it is important to note that the specific mechanisms and performance of these materials may vary depending on their properties, synthesis methods and experimental conditions. Further research is needed to optimize the materials and understand the underlying degradation pathways to advance their practical application in water treatment and environmental remediation.

## 4. Materials and Methods

**Scheme 1:**
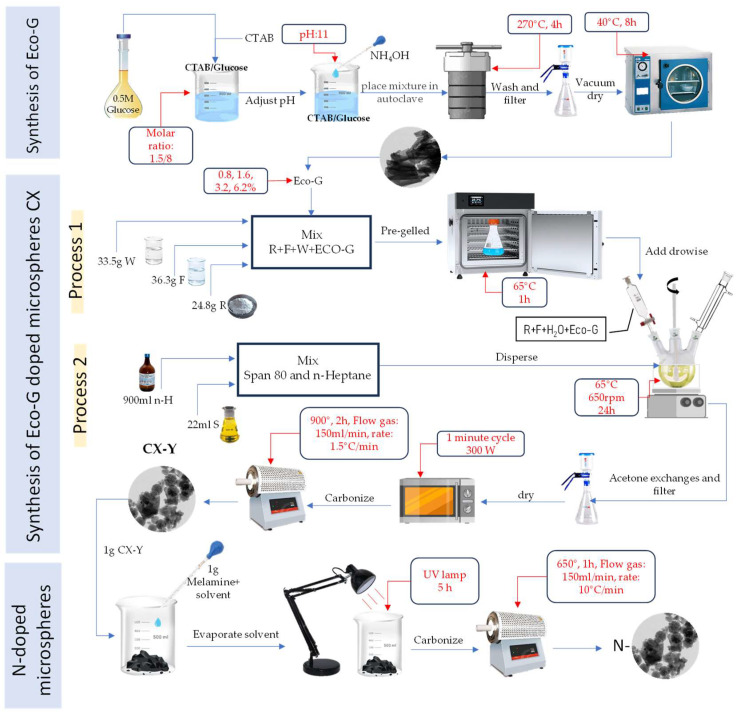
Synthesis process for metal-free catalysts.

The synthesis methodology used to obtain the xerogel microspheres doped with eco-graphene and nitrogen is represented in Scheme 1, and the whole detailed process is explained below:

### 4.1. Synthesis of Eco-Graphene (Eco-EG)

Eco-graphene (Eco-G) was obtained via the controlled hydrolysis of glucose, using CTAB and ammonia as structure-directing agents, followed by a hydrothermal process. For the synthesis, a 0.5 M glucose solution was prepared, and CTAB was dissolved in CTAB/glucose at a molar ratio of 1.5/8. NH_4_OH, which was then added to the mixture at a volumetric ratio of 1/10 to obtain a pH of 11. The mixture was placed in a Teflon-lined autoclave at a volumetric steam/liquid ratio of 3/2, sealed and heat-treated at 270 °C for 4 h in an oven. Subsequently, the autoclaves were slowly cooled to room temperature. The product was filtered and washed with distilled water several times. Finally, the product was dried in a vacuum oven at 40 °C for 8 h.

### 4.2. Synthesis of Eco-G-Doped Microspheres Carbon Xerogels

Carbon xerogel microspheres were synthesized using an emulsion inverse–emulsion reaction of resorcinol (R) and formaldehyde (F) within an organic medium. The process involved creating a water solution of R and F with molar ratios of R/F = 1/2 and R/W = 1/15, e.g., 24.8 g of R, 36.3 g of F and 33.5 g of W. In the same mixture, a percentage of Eco g synthesized previously (0.8, 1.6, 3.2, 6.2%) was added, obtaining different CX-Y materials, where Y is the corresponding percentage of Eco-G. Each mixture was pre-gelled at 65 °C for 1 h. This pre-gelled solution was then added dropwise into a separate solution comprising 22 mL of Span 80 (S) and 900 mL of n-heptane, which was stirred at 650 rpm and refluxed at 65 °C. The R/S molar ratio used in this step was 4.42.

The organic gel was subjected to aging at 65 °C at 650 rpm for 24 h. Afterward, the gel was recovered through filtration and immersed in acetone. The acetone was changed twice daily for five days to facilitate the exchange of water within the pores, aiming to reduce porosity shrinkage during the subsequent drying process and to eliminate the surfactant Span 80. 

In the next step, the xerogel microspheres underwent microwave drying under an argon atmosphere using a Fagor (800 W) microwave oven. An argon flow was introduced through a hole at the back of the microwave during a 1 min cycle at 300 W. This drying process was repeated until a stable weight was achieved without temperature control. 

Finally, the resulting carbon xerogel microspheres were obtained via the carbonization of the organic gel at 900 °C for 2 h under a continuous flow of nitrogen gas (150 mL/min). A low heating rate of 1.5 °C/min was employed to ensure the gentle removal of pyrolysis gases during the carbonization process. 

### 4.3. N-Doped Eco-G-Doped Microspheres Carbon Xerogels

The material doped with the highest percentage of Eco g was taken to obtain an N-doped material; melamine was used as the nitrogen source. Then, 1 g of CX-6.2 was used with 1 g of melamine, resulting in the CX-6.2-NM sample. The functionalization of CX-6.2 was accomplished using the incipient wetness impregnation technique, wherein the appropriate amount of melamine solution was then added gradually to the dried material. The wet solid obtained was left under a UV lamp for 5 h, during which the solvent was evaporated and subsequently removed. The impregnated sample was finally treated at 650 °C for 1 h under a continuous flow of nitrogen gas (150 mL/min) with a heating rate of 10 °C/min.

### 4.4. Textural and Chemical Characterization

The texture and morphology of the samples were analyzed using scanning electron microscopy (SEM) with an FEI microscope model Quanta 400. For a more detailed investigation of the Eco g structure and synthesized a microsphere, high-resolution electron microscopy (HRTEM) was performed using a Thermo Fisher Scientific microscope model Talos F200X.

The textural properties of the samples were evaluated using N_2_ and CO_2_ adsorption at a temperature of −196 °C and 0 °C, respectively. Before the analysis, the samples were subjected to overnight outgassing at 110 °C under vacuum conditions (10^−6^ mbar) to ensure the removal of any adsorbed gases or impurities. The N_2_ adsorption data allowed for the determination of the specific surface area (S_BET_) by applying the Brunauer–Emmett–Teller (BET) method. The micropore volume of W_0_ (N_2_) and W_0_ (CO_2_) and the average micropore volume of L_0_ (N_2_) and L_0_ (CO_2_) were determined by applying the Dubinin–Radushkevich (DR) equation. The total pore volume (V_0.95_) was obtained by measuring the N_2_ adsorption volume at 0.95 relative pressure. Additionally, the mesopore volume (V_MESO_) was calculated as the difference between V_0.95_ and W_0_ (N_2_). 

Raman spectra were acquired using Micro-Raman JASCO NRS-5100 dispersive spectrophotometer, utilizing a 532 nm laser line.

Quantitative chemical analysis of nitrogen, carbon and hydrogen was carried out with a TruSpec Micro-Leco elemental analyzer. X-ray photoelectron spectroscopy (XPS) was performed in a Kratos Axis Ultra-DLD spectrometer equipped with a hemispherical electron analyzer connected to a detector DLD (delay-line detector) and an Al-Kα monochromator with a power of 600 W. The X-ray source is an Mg/Al double anode of 450 W.

### 4.5. O_2_ Reduction Reaction (ORR)

The electrochemical characterization of the materials was conducted using a Biologic VMP Multichannel potentiostat with a rotating ring–disk electrode (RRDE) setup. The RRDE had a 3 mm glassy carbon tip, onto which the carbon sample (20 mL ink) was deposited as the working electrode. A platinum wire served as the counter electrode, while an Ag/AgCl reference electrode was used. To prepare the ink, 5 mg of the carbon sample was dispersed in 1 mL of Nafion solution with a volumetric ratio of 1:9 (Nafion 5% solution water). After depositing the ink, the working electrode was dried under infrared radiation. 

Cyclic voltammetry (CV) experiments were conducted using two different electrolytes, one in an N_2_-saturated 0.1 M KOH solution and the other O_2_-saturated 0.1 M KOH solution. The sweeping potential rate used was 50 mV/s, ranging from 0.4 V to −0.8 V (vs. Ag/AgCl), while the RRDE rotated at 1000 rpm. To assess the catalytic performance and determine the onset potential, linear sweep voltammetry (LSV) experiments were carried out in O_2_-saturated 0.1 KOH solution at various rotation rates, raining from 50 rpm to 3500 rpm. The LSV experiments were conducted in a working window from 0.4 V to −0.8 V (vs. Ag/AgCl) at a sweep rate of 5 mVs^−1^. 

To gain an insight into the electron transfer mechanism and kinetics at the electrode, fitting the LSV by mean of the Koutecky–Levich model was employed to determine the number of electrons transferred (n) and the kinetic density current (J_k_). On the other hand, the currents of the platinum ring measured during the LSV experiments permit the calculation of the overall electron transfer number (n) and the percentage of hydrogen peroxide produced (H_2_O_2_%); Equations (5) and (6) were used, respectively.

(5) $$n=\frac{4\cdot I_{D}}{I_{D}-\frac{I_{R}}{N}}$$

(6) $$H_{2}O_{2}\%=100\cdot \frac{2\cdot \frac{I_{R}}{N}}{I_{D}-\frac{I_{R}}{N}}$$

where I_R_ and I_D_ represent the ring and the disk currents, respectively, and N = 0.245 is the collection efficiency of the RRDE.

### 4.6. Electro-Fenton Tests

First, the preparation of the Electro-Fenton electrodes was carried out by preparing a homogeneous paste of finely milled carbon xerogel spheres and polytetrafluoroethylene (PTFE) binder in a mass ratio of 9:1, dried at 80 °C overnight. Then, 40 mg of the resulting paste was weighed, and an area of 2.5 cm × 1 cm was coated on graphite paper.

In the evaluation of the dual-functional electrocatalysts in Electro-Fenton (EF) tests, tetracycline (TTC) was chosen as the emerging pollutant. The system employed was a three-electrode glass cell controlled using a Biologic VMP multichannel potentiostat (BioLogic Science Instruments) using Ag/AgCl as the reference electrode and a Pt wire as the counter electrode. Before carrying out the Electro-Fenton tests, it was ensured that the adsorption effect of the contaminant on the materials was eliminated. For this purpose, the electrode was saturated in the dark for 24 h in the solution composed of (0.5 M Na_2_SO_4_)/TTC to obtain a final concentration of 15 ppm measured at 358 nm using a UV-spectrophotometer model UV-2600i Shimadzu. Then, the EF experiment commenced by applying a voltage of −0.6 V in the potentiostatic mode while simultaneously bubbling O_2_ gas into the solution. To ensure the saturation of the solution, O_2_ gas bubbled for 30 min before the experiment began and continued throughout the experiment’s duration. During the EF experiment, 1 mL aliquots were periodically taken from the glass cell at specified time intervals. The concentration of TTC in each aliquot was immediately analyzed using a UV-spectrophotometer.

## Figures and Tables

**Table 1:** Porous textural properties of the pure, eco-graphene-doped and N-doped carbon xerogels.

Sample	N_2_-Isotherm					CO_2_-Isotherm	
	S_BET_	W_0-N2_	L_0-N2_	V_0.95_	V_meso_	W_0-CO2_	L_0-CO2_
	m^2^g^−1^	cm^3^g^−1^	nm	cm^3^g^−1^		cm^3^g^−1^	nm
CX-B	514	0.21	1.14	0.25	0.04	0.27	0.57
CX-0.8	548	0.22	0.88	0.33	0.09	0.26	0.57
CX-1.6	529	0.21	0.80	0.33	0.09	0.26	0.57
CX-3.2	531	0.22	0.89	0.33	0.12	0.25	0.57
CX-6.2	549	0.22	0.89	0.42	0.20	0.27	0.59
CX-6.2-NM	265	0.11	1.56	0.31	0.24	0.27	0.58

**Table 2:** Analysis of the elemental composition.

Sample	Elemental Composition by EA (wt%)		
	C	H	N
CX-B	96.02	0.30	0.00
CX-0.8	95.60	0.34	0.07
CX-1.6	97.30	0.30	0.07
CX-3.2	95.00	0.33	0.14
CX-6.2	96.16	0.32	0.20
CX-6.2-NM	91.11	0.15	5.27

**Table 3:** Surface chemical composition determined via XPS.

Sample	C_1s_			O_1s_		N_1s_		C_XPS_ (%)	O_XPS_ (%)	N_XPS_ (%)
	BE (eV)	% Peak	Assign.	BE (eV)	% Peak	BE (ev)	% Peak			
CX-B	284.6	58.5	C=C	532.3	50.7			96.3	3.7	-
	285.3	22.8	C–C	533.7	49.3					
	286.6	8.0	C–O							
	288.2	4.0	C=O							
	289.9	4.5	COO−							
	291.7	2.1	π−π*							
CX-6.2-NM	284.6	55.0	C=C	530.8	12.0	398.4	51	92.7	2.3	5.0
	285.3	23.7	C–C	532.3	50.4	399.8	33			
	286.6	9.5	C–O	533.5	37.7	401.0	16			
	288.0	5.0	C=O							
	289.9	4.1	COO−							
	291.5	2.7	π−π*							
Eco-G	284.5	72.8	C=C	530.6	30.9	398.4	47.4	75.7	11.3	13.1
	285.7	20.9	C–C	532.0	69.1	399.6	47.2			
	286.9	6.3	C–O			401.8	5.4			

**Table 4:** Electrochemical parameters obtained from the analysis of LSV curves.

Sample	E_onset_ (V)	^a^ J_k_	n
		mAcm^−2^	
CX-B	−0.19	2.9	3.3
CX-0.8	−0.18	6.6	3.6
CX-1.6	0.16	7.4	3.3
CX-3.2	−0.17	9.3	3.0
CX-6.2	−0.18	9.6	3.5
CX-6.2-NM	−0.15	13.1	3.4
ECO-G	−0.26	2.1	1.5

^a.^ J_k_ and n refer to K-L fitting at −0.6 V (vs. Ag/AgCl).

## Data Availability

Not applicable.
